# High-Performance Photo-Modulated Thin-Film Transistor Based on Quantum dots/Reduced Graphene Oxide Fragment-Decorated ZnO Nanowires

**DOI:** 10.1007/s40820-016-0083-7

**Published:** 2016-03-05

**Authors:** Zhi Tao, Yi-an Huang, Xiang Liu, Jing Chen, Wei Lei, Xiaofeng Wang, Lingfeng Pan, Jiangyong Pan, Qianqian Huang, Zichen Zhang

**Affiliations:** 1 School of Electronic Science and Engineering, Southeast University, Nanjing, 210096 People’s Republic of China; 2 State Key Laboratory of Precision Measurement Technology and Instruments, Collaborative Innovation Center for Micro/Nano Fabrication, Device and System; Department of Precision Instrument, Tsinghua University, Beijing, 100084 People’s Republic of China; 3 School of Information and Communication Engineering, Beijing University of Posts and Telecommunications, Beijing, 100876 People’s Republic of China; 4 Institute of Semiconductors, Chinese Academy of Science, Beijing, 100083 People’s Republic of China

**Keywords:** Thin-film transistor, Quantum dots, Reduced graphene oxide, ZnO nanowires

## Abstract

In this paper, a photo-modulated transistor based on the thin-film transistor structure was fabricated on the flexible substrate by spin-coating and magnetron sputtering. A novel hybrid material that composed of CdSe quantum dots and reduced graphene oxide (RGO) fragment-decorated ZnO nanowires was synthesized to overcome the narrow optical sensitive waveband and enhance the photo-responsivity. Due to the enrichment of the interface and heterostructure by RGO fragments being utilized, the photo-responsivity of the transistor was improved to 2000 A W^−1^ and the photo-sensitive wavelength was extended from ultraviolet to visible. In addition, a positive back-gate voltage was employed to reduce the Schottky barrier width of RGO fragments and ZnO nanowires. As a result, the amount of carriers was increased by 10 folds via the modulation of back-gate voltage. With these inherent properties, such as integrated circuit capability and wide optical sensitive waveband, the transistor will manifest great potential in the future applications in photodetectors.

## Introduction

In the last few years, photo-modulated thin-film transistors (TFTs) have found widespread applications in photo-sensitive materials [1–3] such as in the electronic and electro-optic components. For applications in integrated circuit [4, 5], amorphous metal oxide semiconductor (AMOS)-based TFTs have been utilized in phototransistors and photo-sensors due to their high sensitivity, electron mobility, and on/off ratio. Because of the wide band gap (~3.3 eV) and a large exciton binding energy (60 meV), ZnO not only can be considered as a promising candidate for ultraviolet (UV) photo-sensors but also can be used as an active layer for photo-modulated TFTs [6–8]. Due to the rapid development of tunable semiconductor quantum dots (QDs), they have been focused on applications in optoelectronics devices, such as light emitting devices and photodetectors [9–12]. By taking into consideration of the recent research work in optoelectronics devices, the size-tunability of the QDs can be useful in detecting more regions of optical spectrum [13].

Compared with the traditional photo-modulated transistor, as reported by Yuyu Bu [14], photo-sensitive waveband and fast response speed can be improved by using hybrid materials containing ZnO nanowires and QDs. However, due to the mechanism of carrier transfer, low photo-responsivity was still a challenge in photo-modulated TFT application [15]. In order to improve the performance of the hybrid materials containing ZnO nanowires and QDs, graphene was exploited. Graphene as an atomic layer with remarkable electric and optical properties was considered as a highly desired material in applications in photodetectors, biological imaging, and telecommunication system [16–18]. Based on its linear electronic dispersion and the electrons transferred along the surface, graphene can be used as an efficient intermediary material for injected electrons. In addition, high drift velocity of the charge in graphene also allows for more efficient separation of electron from the site of injection [19–22]. Compared with the monolayer graphene, reduced graphene oxide (RGO) with similitude electric properties can be utilized to provide defect energy states [23, 24]. In addition, due to the existence of narrow photo-sensitive waveband and discontinuity distribution, hybrid materials containing ZnO nanowires/RGO fragments and QDs/RGO fragments were not recommended for use in photo-modulated transistors [25].

In this paper, a photo-modulated transistor with TFTs structure is demonstrated using CdSe QDs/RGO fragments decorated on the surface of ZnO nanowires as the active layer [16]. CdSe QDs, as an electron donor, was attached to the RGO fragments to expand the photo-sensitive waveband. RGO fragments were exploited to provide a favorable photo-responsivity. Eventually, the characteristics, such as photocurrent, responsivity, and rectifying capability, and the performances of this device were also measured and analyzed.

## Experiment Sections

### Fabrication of ZnO Nanowires

**Fig. 1:**
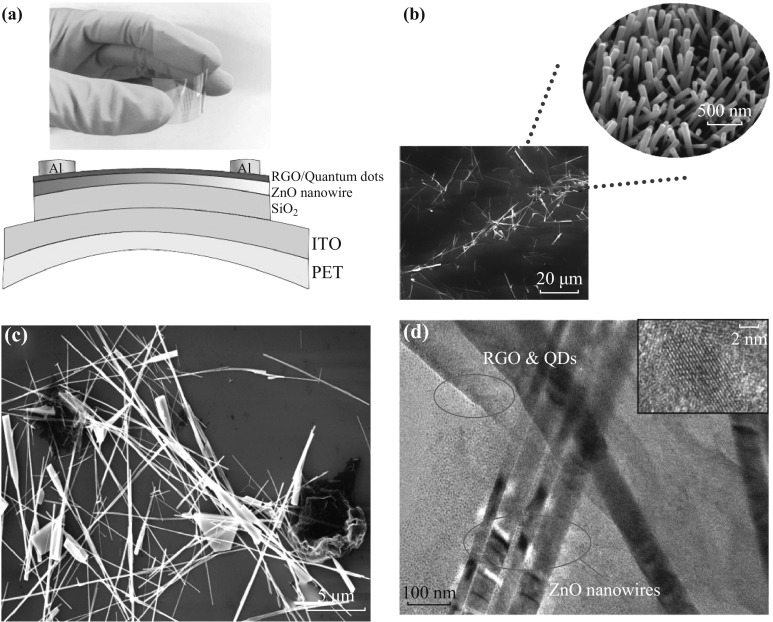
Characterization of flexible photo-modulated transistors. **a** The structure of the CdSe QDs/RGO decorated on the surface of ZnO nanowires photo-modulated transistor. **b** SEM image of ZnO nanowires. **c** SEM image of hybrid materials. **d** TEM image of the CdSe QDs/RGO/ZnO nanowires and CdSe QDs *inset*

The CdSe QDs/RGO fragment-decorated on the surface of ZnO nanowires was utilized as the active layer and photo-sensitive layer (Fig. 1a). The fabrication process of ZnO nanowires was illustrated in the following: First, a piece of silicon (001) wafer was washed sequentially with acetone, ethanol, and deionized water for 10 min, respectively. The quartz boat filled with 0.2 g zinc powder was covered by the silicon wafer before being transferred to the chamber. With a flow rate of 20 sccm (Ar_2_:O_2_ = 3:1), the pressure of chamber under 7.5 × 10^−3^ torr and controlled temperature of plasmon-enhanced chemical vapor deposition (PECVD) of 800 °C, high-quality ZnO nanowires with a length of 25 μm and a diameter of 150 nm was obtained. The aspect ratio is higher than 150 as shown in Fig. 1b.

### Synthesis of CdSe QDs/RGO Fragments

Graphene oxide (GO) was purchased from Hengqiu Graphene Technology (Suzhou Co. Ltd.). It was made to react with a reducing agent NaBH_4_ to produce the RGO fragments of ~5 μm in diameter. N-type CdSe QDs modified with tri-n-octylphosphine oxide (TOPO) of ~7 nm in diameter were utilized as the photo-sensitive material [purchased from Mesolight Inc (Suzhou Co. Ltd)]. The hybrid CdSe QDs/RGO fragment solution consisted of 5 mg mL^−1^ CdSe QDs and 1 mg mL^−1^ RGO solution. In order to obtain CdSe QD-decorated RGO fragments, hybrid solution was then oscillated in ultrasonic bath before it was filtered by the membrane material.

### Manufacture of the Device

The three-terminal gated photo-modulated transistor was fabricated on the substrate of polyethylene terephthalate (PET). The PET coating with ITO layer and SiO_2_ insulating layer was deposited by magnetron sputtering process. After ZnO nanowires were dispersed in ethanol, the dispersion solution was spin-coated on the SiO_2_ substrate at a speed of 2000 r min^−1^. Afterward, the hybrid solution of CdSe QDs/RGO fragments was spin-coated on ZnO nanowire film at a speed of 1000 r min^−1^. Finally, thermal annealing was carried out at a temperature of 180 °C to promote uniformity of hybrid material layer and remove the solvent before the electroplating process with the mask (W:L = 100 μm:20 μm) for the electrodes. The channel of the device was characterized by scanning electron microscope (SEM, FEI Quanta 200, Holland) and the hybrid material samples were characterized by transmission electron microscope (TEM, JEM-2100, Japan). The morphology of the hybrid material containing RGO and ZnO nanowires is shown in Fig. 1c. It can be seen that ZnO nanowires of ~ 150 nm in diameter and ~25 µm in length are covered by RGO fragments and are thus in close contact with the RGO fragments. In the inset of Fig. 1d are shown the diameters of CdSe QDs, which are of ~7 nm, with clear crystal lattices. In addition, the clear crystal lattices demonstrate the high crystallization of the CdSe QDs.

## Results and Discussion

From Fig. 1d, it can be seen that the size of RGO fragments is much larger than that of CdSe QDs. The CdSe QDs were only distributed on the surface of RGO fragments. This confirms that CdSe QDs were blended with RGO fragments and the contact is constructed between RGO fragments and ZnO nanowires.

**Fig. 2:**
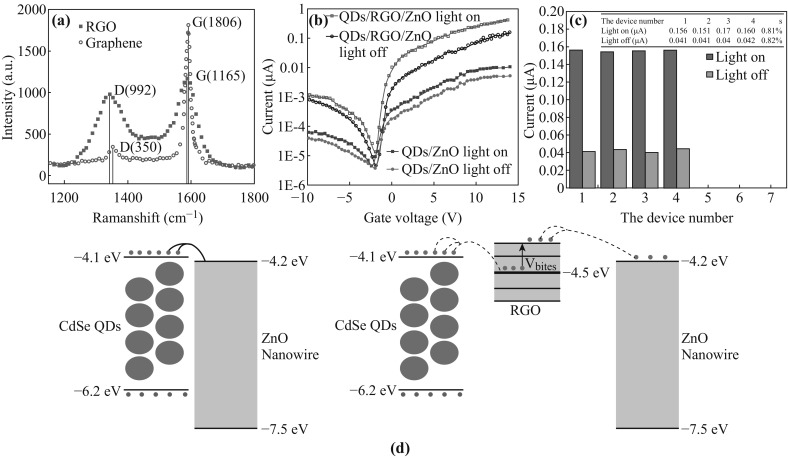
**a** The Raman spectrum of graphene and RGO. **b** Photocurrent variation of two different devices with and without incident light (*V*_DS_ = 5 V, *λ* = 580 nm). **c** The reproducibility test of different devices (*V*_DS_ = 5 V, *V*_GS_ = 8 V, *λ* = 580 nm). **d** Energy band schematic of the devices with and without using RGO

Figure 2a shows the Raman spectrum (carried on Renishaw, England) of the graphene and RGO, where the peak intensities of *D* band and *G* band are, respectively, located at 1350 and 1580 cm^−1^ for RGO. It is clearly evident that the defect states of RGO existed because the peak intensities of *D* band and *G* band shifted [26, 27].

The dimension of channel area is 100 × 20 μm^2^. When the incident power intensity of 10 μW cm^−2^ is applied, the photo-responsivity was recorded, which can be calculated using Eq. (1) [25], and the transfer characteristic curve is shown in Fig. 2b.

1 $$R = \frac{{I_{\text{total}} - I_{\text{dark}} }}{P} = \frac{{I_{\text{ph}} }}{\rho S},$$

where *P* represents the optical power, *I*_total_ denotes the total current, *I*_dark_ is the dark current, *I*_ph_ implies the photocurrent, ρ indicates the optical incident power density, and *S* is the effective area for photo-electric reaction, respectively. When the drain–source voltage of 5 V is applied and the wavelength of incident light is 580 nm, the responsivity of the CdSe QDs/RGO/ZnO nanowires calculated was of ~2000 A W^−1^, which is approximately 2 orders of magnitude larger than that of CdSe QDs/ZnO nanowires. Four devices have been assembled and measured at the same conditions with the drain–source voltage being 5 V and back-gate voltage being 8 V, respectively. The standard deviation of the photocurrent shown in Fig. 2c is found to be below 1 % when the incident wavelength is at 580 nm.

The energy band schematics of two kinds of devices are illustrated in Fig. 2d. The charge transfer takes place across the interfaces of CdSe QDs/RGO/ZnO nanowires. In the CdSe QDs/ZnO nanowire hybrid materials, CdSe QDs are regarded to possess the optical property, where carriers can be generated and transferred from the ligand-capped CdSe QDs to the ZnO nanowires. Before the recombination of the electron–hole pairs in CdSe QDs happens, the charge can be injected into ZnO nanowire (*E*_CB_ = −4.2 eV, *E*_VB_ = −7.7 eV) and then drifted to the source electrode under the bias. In the meanwhile, an equal number of carriers can be provided by the drain electrode to satisfy the conversation of charge in the channel (Fig. 2d, left). Since vacuum energy level is considered as the reference of potential energy, valence band (*E*_VB_) and conduction band (*E*_CB_) of CdSe QDs can be also measured, which were −4.1 and −6.2 eV. The Fermi level of graphene was reported to be −4.5 eV [19], which is much lower compared with the conduction band of CdSe QDs (Fig. 2d, right). Therefore, after incorporating with RGO, electrons can be transferred more efficiently from the conduction band of CdSe QDs to graphene due to the more favorable energy barrier between the interface of CdSe QDs and RGO. Furthermore, the electrons can jump more efficiently from the Fermi level of the graphene to the defect level and transfer to the ZnO nanowires when the electrical field is constructed by the positive back-gate voltage directed to source electrode. Consequently, due to the more favorable energy of CdSe QDs/RGO/ZnO hybrid, it can effectively promote the transfer of charge from CdSe QDs to ZnO nanowire. Moreover, fewer traps existed at the interfaces across CdSe QDs/RGO fragment/ZnO nanowire hybrid. As a result, electron annihilation is decreased and the carrier separation in hybrid material is benefited. Based on Fig. 2b, due to the enrichment of the interface and heterostructure by RGO fragments being utilized, the photocurrent has been improved approximately 100 folds. According to Eq. (1), the corresponding photo-responsivity of the transistor with RGO fragments is induced by approximately 100 folds.

**Fig. 3:**
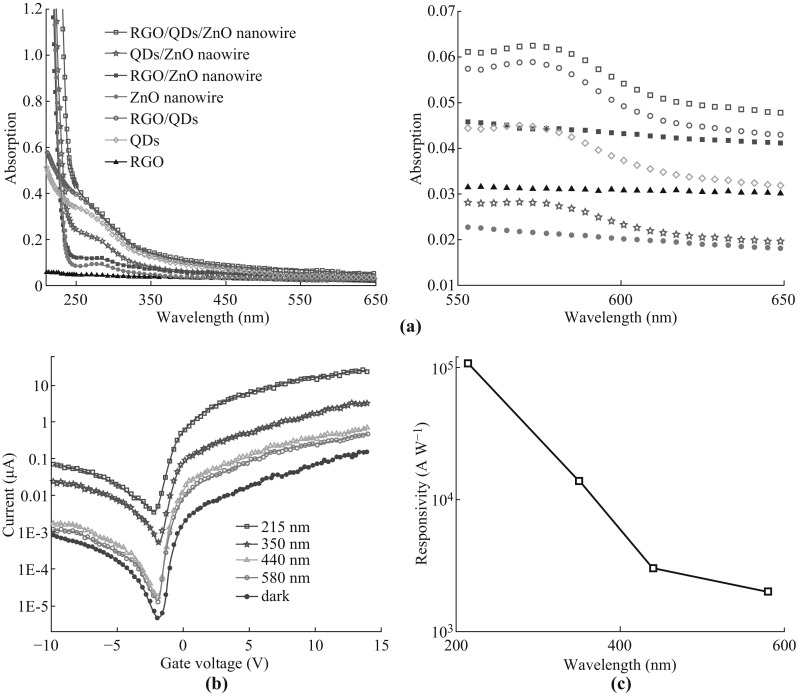
**a** Absorption spectra of CdSe QDs, RGO, ZnO nanowires, and the hybrid materials. **b** Transfer character curves for CdSe QDs/RGO/ZnO nanowire exposed to different wavelengths of incident light. **c** The responsivity curve for different devices at different wavelengths of incident light (*λ* = 215, 350, 440, 580 nm)

To investigate the optical absorption characteristics of CdSe QDs, RGO fragments, ZnO nanowires, and CdSe QDs/RGO/ZnO nanowire hybrids, absorption spectra are respectively recorded and characterized in Fig. 3a. Compared with the optical absorption spectra of CdSe QDs/ZnO nanowire hybrid, the optical absorption intensity is enhanced for CdSe QDs/RGO/ZnO nanowires at wavelengths from 200 to 650 nm. Meanwhile, it can be proved that the optical absorption can be increased for the visible-light photo-modulated TFTs based on CdSe QDs/RGO/ZnO nanowires with excitonic transition peak at wavelength of 580 nm (Fig. 3a).

Figure 3b illustrates the transfer characteristic curves of CdSe QDs/RGO/ZnO nanowires device in the wavelength ranging from 215 to 580 nm. The result indicates that there occurred a decrease of current, which fluctuated, from ultraviolet to red. Traditionally, the balance between the number of the electrons and holes is maintained and the material thus remains electrically neutral with the minimum current when the gate voltage equalled to 0 V. However, the electrons are injected into the RGO/ZnO nanowires by the built-in field when N-type CdSe QDs are utilized as the electron donor. In order to achieve the electric neutrality, a certain amount of holes are ought to be provided from the RGO/ZnO nanowires when N-type QDs are doped in the hybrid material. Therefore, the negative back-gate voltage plays an important role in attracting holes and achieving the balance between the electrons and holes when the current is minimum. Additionally, photogenerated carriers are consistent with the results shown in Fig. 3a, where the photocurrent is relatively at a high value near the wavelength of excitonic transition peak. As illustrated in Fig. 3c, the photoresponse waveband of the hybrid material can be expanded and the opportunity for the exciton separation can be improved. This contributes to the enhanced photocurrent and photo-responsivity gain.

**Fig. 4:**
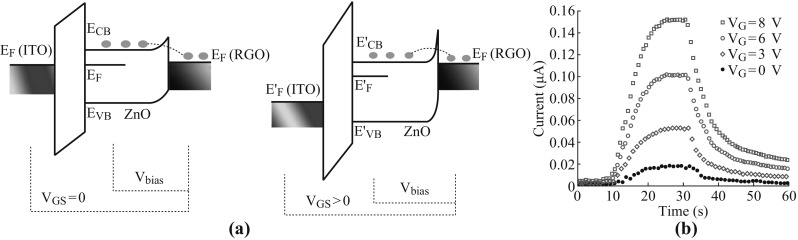
**a** Schematic diagram of the bias mechanism of the device with positive back-gate voltage on and off. **b** Time-dependent photocurrent response with different gate voltages (*V*_DS_ = 5 V) under illumination (580 nm)

The photo-responsivity can be enhanced by modulating the back-gate voltage due to the existence of carrier transport mechanism [22, 28, 29] (in Fig. 4a, left). It shows the energy band distribution with adjusting the diverse voltages of *V*_GS_. For the interface of RGO/ZnO nanowires near the source electrode, a Schottky barrier is formed between ZnO nanowires and RGO fragments. When *V*_bias_ is applied in the ZnO nanowires and RGO fragments near the source electrode, electrons in the RGO fragments can be transferred to the ZnO nanowires. Thus, *I*_S_ increased as the bias voltage is increased. In addition, *E*_F(RGO)_ and *E*_F(ZnO)_ levels are shifted upwards as *V*_GS_ is above zero. In the meanwhile, the width of Schottky barrier is reduced simultaneously (Fig. 4b, right). When the photo-modulated transistor is excited by the incident light (*λ* = 580 nm), the increment of the photocurrent can be measured by applying the back-gate voltage as demonstrated in Fig. 4b. It was found that the value of the applied back-gate voltage is proportional to the photocurrent. For instance, the photocurrent reached a value of 0.15 μA when the back-gate voltage of 8 V was applied. And the photocurrent values of 0.015, 0.05, and 0.10 μA were measured when the back-gate voltages were 0, 3, and 6 V, respectively. Consequently, the electrons which tunneled from RGO fragments to ZnO nanowires were easily obtained by applying the positive back-gate voltage, and this led to 10-fold increase in the photo-responsivity.

## Conclusions

In summary, a novel photo-modulated transistor based on the TFT structure was fabricated by means of hybrid material containing CdSe QDs/RGO fragments and ZnO nanowires. By incorporating the RGO fragment, the interface and heterostructure of this hybrid material were improved and the photo-responsivity of this transistor was improved by ~10^2^ times. This novel photo-modulated transistor with hybrid materials was found to be more advantageous than that with CdSe QDs/ZnO nanowires in the visible incident light. In addition, the photocurrent of this device was improved by 10 times by manipulating the back-gate voltages. The photo-responsivity (2000 A W^−1^) was also enhanced and the photoresponse waveband was upgraded. Since the process of our experiment was operated at room temperature, the advantages of using hybrid materials in photo-modulated transistor suggest that the transistor can be a potential candidate for applications in the large-area transparent flexible photo electronics.
